# Generalizing Boltzmann Configurational Entropy to Surfaces, Point Patterns and Landscape Mosaics

**DOI:** 10.3390/e23121616

**Published:** 2021-12-01

**Authors:** Samuel A. Cushman

**Affiliations:** USDA Forest Service, Rocky Mountain Research Station, Flagstaff, AZ 86001, USA; samuel.cushman@usda.gov

**Keywords:** Boltzmann entropy, landscape, configuration, thermodynamics, complexity, pattern, scale

## Abstract

Several methods have been recently proposed to calculate configurational entropy, based on Boltzmann entropy. Some of these methods appear to be fully thermodynamically consistent in their application to landscape patch mosaics, but none have been shown to be fully generalizable to all kinds of landscape patterns, such as point patterns, surfaces, and patch mosaics. The goal of this paper is to evaluate if the direct application of the Boltzmann relation is fully generalizable to surfaces, point patterns, and landscape mosaics. I simulated surfaces and point patterns with a fractal neutral model to control their degree of aggregation. I used spatial permutation analysis to produce distributions of microstates and fit functions to predict the distributions of microstates and the shape of the entropy function. The results confirmed that the direct application of the Boltzmann relation is generalizable across surfaces, point patterns, and landscape mosaics, providing a useful general approach to calculating landscape entropy.

## 1. Introduction

The calculation of configurational entropy of landscapes has emerged as a topic of considerable recent interest, as appreciation of the connections between thermodynamics and landscape processes becomes more apparent [1,2]. Several recent methods have been proposed to calculate configurational entropy, based on Boltzmann entropy. The Cushman [3,4] method directly applies the classic and iconic Boltzmann relation (s = klogW) to spatial patterns. The Boltzmann relation states that the entropy of a system is proportional to the logarithm of the number of macrostate, or unique configurations, that produce the observed macrostate, or global system property. Other authors have proposed more complex solutions based on multiresolution analysis [5,6] and Wassenstein entropy [7,8]. There has yet to be a formal comparison of the relationships among these different methods and their consistency with fundamental thermodynamic principles. Recently, Gao and Li [9] showed that modification of the Gao et al. [5,6] multiresolution approach was mostly thermodynamically consistent and applicable to both patch mosaics and surface patterns. Cushman [10] (this volume) showed that the Cushman method of directly applying the Boltzmann relation to calculate configurational entropy through spatial permutation and counting the number of arrangements (microstates) that produces the same total edge length (macrostate) of a landscape mosaic lattice was fully thermodynamically consistent. The goal of this paper is to show that the Cushman method is also fully generalizable to surfaces, point patterns, and landscape mosaics.

The Boltzmann relation is potentially a foundational method for computing configurational entropy for several reasons. First and foremost, it is the original, classic, and iconic formulation expressing the entropy of the system in terms of the arrangement of its particles. The concept of entropy being proportional to the logarithm of the number of microstates in a macrostate is a transformative idea in science. It provides an elegant and profound understanding of order and disorder in relation to the probability distribution of different states. Second, as a framework for computing the configurational entropy of landscapes, the Boltzmann relation is particularly appropriate given the direct analogies between particular arrangements of landscape elements and microstates in the Boltzmann system and measured spatial attributes of a landscape pattern as the Boltzmann macrostate. Cushman [3,4] applied this to landscape mosaics. The goal of this paper is to show that the direct application of the Boltzmann relation for computing landscape entropy also applies in the exact same way to measuring the entropy of point patterns and surfaces. This is important, in that showing that the elegant and simple Boltzmann relation is also generalizable to all kinds of spatial patterns provides a strong unification of theory with methods for ecological and spatial thermodynamics.

The essence of the Cushman [3,4] approach is to apply the Boltzmann relation directly to spatial patterns of a landscape mosaic of categorical patches. This is done in three steps. First, a landscape mosaic is spatially permuted a large number of times, producing a distribution of randomized patterns (random shuffling of the locations of pixels while not changing their values). Then the total edge length (the linear distance along edges of pixels where two different cover classes touch) is calculated for the actual landscape and the large number of permuted patterns. Next, following the Central Limit Theorem, Cushman [4] showed that the edge lengths of the permuted landscapes (microstates) are normally distributed. A Gaussian function is fit to this distribution. Cushman [4] also showed that the entropy function resulting from this distribution is parabolic, given that the logarithm of a normal distribution is a parabola, and entropy is proportional to the logarithm of the number of microstates producing a given macrostate.

Cushman [10] showed that application of the Cushman approach to calculating the Boltzmann entropy of a landscape mosaic was fully thermodynamically consistent in that: (1) the distribution of microstates was indeed Gaussian; (2) the entropy function was parabolic; (3) the mean value of the Gaussian distribution of microstates was a linear function of landscape dimensionality; (4) the standard deviation of the Gaussian distribution of microstates was a power function of landscape dimensionality with power equal to exactly 0.5; (5) the entropy of a pattern is maximal in a state of spatial randomness and is lower when the pattern is both aggregated and when it is dispersed; (6) the entropy increases in random mixing experiments toward maximum entropy achieved at fully spatial randomness; and (7) at maximum entropy a random mixing experiment maintains the landscape in the region of maximum entropy (full spatial randomness).

These results seem to suggest that the simple, direct application of the classic Boltzmann relation to calculating landscape entropy is theoretically fully consistent with classic formulations of Boltzmann entropy with no modification, and is fully thermodynamically consistent. To be a fully generalized method, however, the approach must be shown to be applicable to all kinds of spatial patterns, including surfaces and point patterns, in addition to landscape mosaics. This paper presents simulations to show that the Cushman approach to calculating Boltzmann entropy is equally applicable to surfaces, point patterns, and landscape mosaics, and in each case is fully thermodynamically consistent.

## 2. Methods

**Figure 1:**
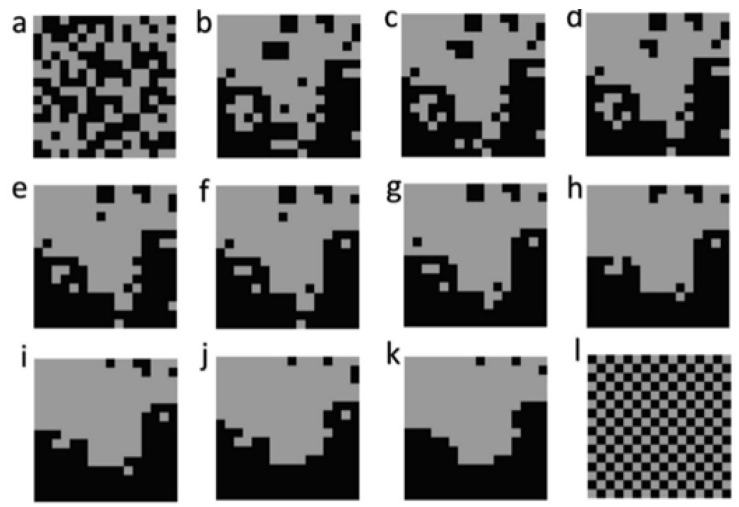
The 12 test landscapes. (**a**) spatially random; (**b**) H1; (**c**) H2; (**d**) H3; (**e**) H4; (**f**) H5; (**g**) H6; (**h**) H7; (**i**) H8; (**j**) H9; (**k**) H10; (**l**) checkerboard.

I build on the simulation framework used in [4] in which I used a neutral landscape model [11] to generate fractal landscape mosaics that control the dimensionality of the landscape, the number of cover classes, and the degree of spatial aggregation. Specifically, I chose a landscape dimension of 16 × 16 pixels, two cover classes, each with 50% coverage. For that landscape composition, I varied configuration by controlling the H parameter of QRULE, which controls fractal aggregation. H increases from 0 for unaggregated patterns to 1 for highly aggregated patterns. In [4] I varied the H parameter across 10 levels, from 0.1 to 1 by steps of 0.1 (Figure 1). I also produced a fully spatially random map and a fully dispersed map (checkerboard pattern).

**Figure 2:**
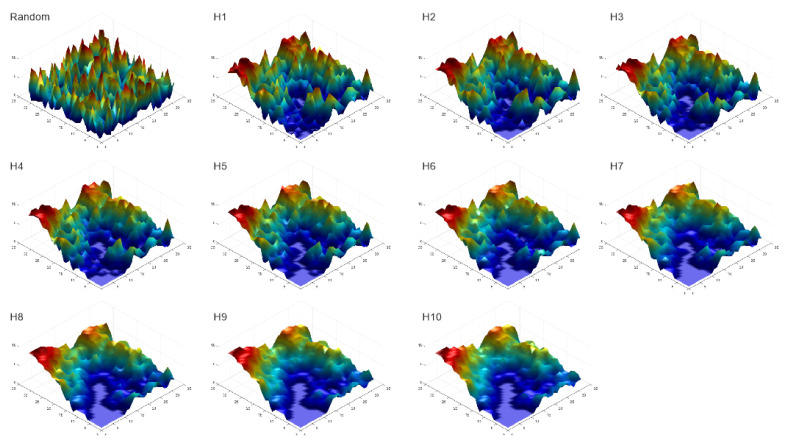
Visualization of surface patterns across a gradient from full spatial random arrangements of pixels through ten steps of the H aggregation parameter (0.1 to 1.0).

In this generalization of the Cushman method of calculating Boltzmann configurational entropy, I use landscape dimensionality of 32 × 32 pixels across the same levels of H (10 steps from 0.1 to 1.0). Instead of a binary, two class landscape, however, I use QRULE to generate surfaces and point patterns. To generate a surface, I specified 10 cover types each with 10% cover extent. This produces a surface which has the same fractal aggregation as the binary map, but in which the 10 cover types represent sequential “heights” of the surface (Figure 2).

**Figure 3:**
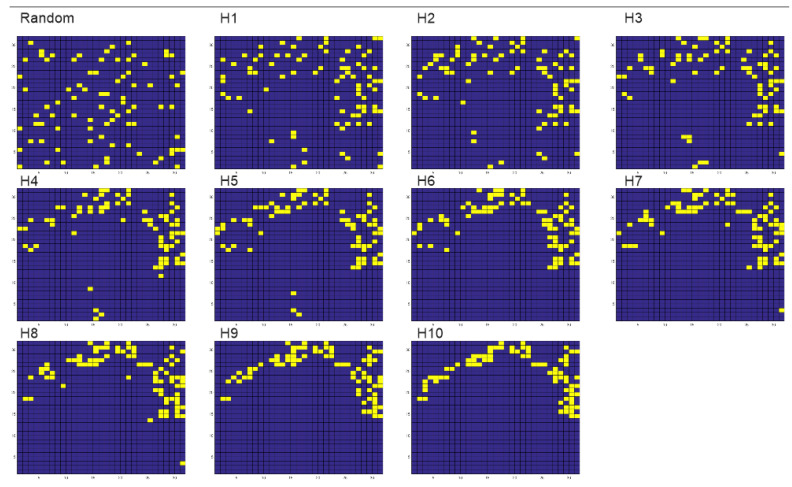
Visualization of point patterns across a gradient from full spatial random arrangements of pixels through ten steps of the H aggregation parameter (0.1 to 1.0). Point locations are specified by yellow cells on a blue background lattice.

Point patterns were similarly generated using the H parameter in QRULE. This was done by selecting the centroids of pixels with value 10 from the multiclass fractal maps used to produce surfaces. This produces fractal gradient of point aggregation across the 10 steps of H aggregation parameter from 0.1 to 1.0 (Figure 3).

**Figure 4:**
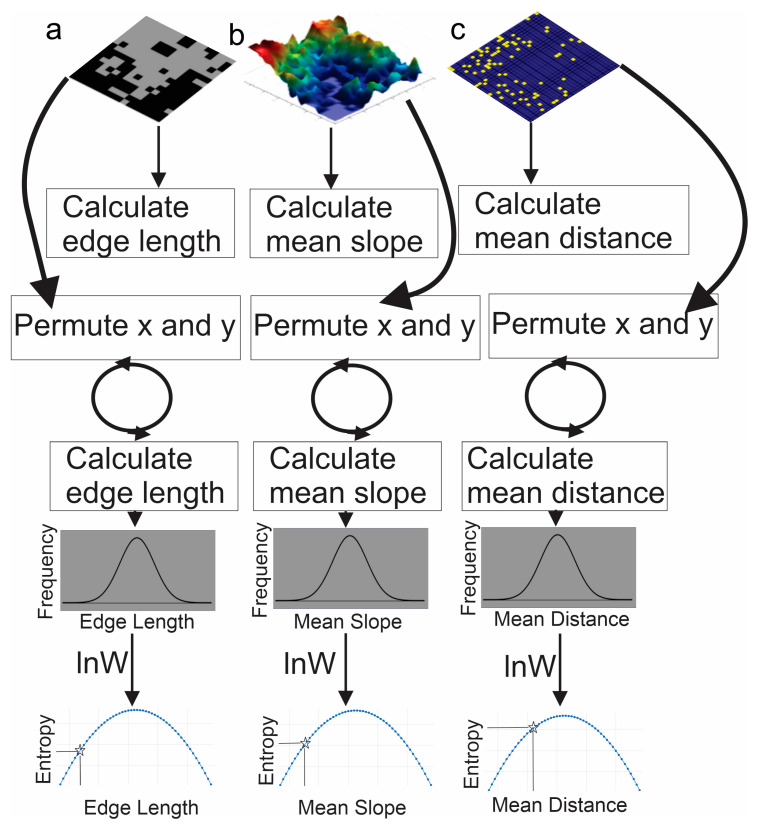
Schematic showing the analytical consistency of applying the Boltzmann relation to calculate configurational entropy for landscape mosaics (**a**), surfaces (**b**), and point patterns (**c**). There are six main steps that are identical between the three methods: (1) obtain a landscape map (either a mosaic, a surface or a point pattern); (2) calculate the state variable (edge length for a mosaic, mean slope for a surface, mean distance between points for a point pattern); (3) permute the map, recalculating the state variable a large number of times; (4) fit a normal probability function to the permuted distributions; (5) calculate the logarithm of the fitted normal probability function, which is the “entropy function”; and (6) find the value of entropy (y axis) that corresponds to the observed value of the objective parameter.

The Cushman approach to directly apply the Boltzmann relation to calculate configurational entropy is highly consistent when applied to landscape mosaics, surfaces, and point patterns (Figure 4). The approach works in six steps: (1) obtain a landscape map (either a mosaic, a surface or a point pattern); (2) calculate the state variable (edge length for a mosaic, mean slope for a surface, mean distance between points for a point pattern); (3) permute the map, recalculating the state variable a large number of times; (4) fit a normal probability function to the permuted distributions; (5) calculate the logarithm of the fitted normal probability function, which is the “entropy function”; and (6) find the value of entropy (y axis) that corresponds to the observed value of the state variable in the sample landscape.

The application of the Cushman [4] method to calculate configurational entropy to a landscape lattice mosaic is based on the value of edge length between dissimilar classes of pixels. An analogous measure to generalize this to surfaces is the local slope, which is conceptually equivalent to an “edge” on a surface. Therefore, for each surface pattern and the permuted distributions of them I computed the local slope as the average absolute difference in value (height) between each cell and its four orthogonal neighbors. In other words, to calculate local slope I calculated the average of the absolute differences between each focal cell and its four orthogonal neighbors. This is the average slope (delta elevation/delta distance) in the four directions around each focal cell. This produces a measure of the total “slope” of the surface at the scale of neighboring pixels. To generalize to point patterns in a conceptually consistent way I computed the mean distance between all pairs of points. This measure computes the average distance between pairs of points which is analogous to the mean slope of a surface number.

Following the same procedures as in [4], I spatially permuted the surfaces (Figure 2) and point patterns (Figure 3) a large number of times (1,000,000), each time recalculating the local slope for each pixel (for surface analysis) and mean distance between points (for point patterns). I then used the same procedure as [4] to fit a normal distribution of microstates across values of the macrostate (average slope or mean distance). I then computed the entropy function for both surface and point patterns and plotted the distribution of entropies of the 11 example landscapes (spatially random, H1…H10). For both surfaces and point patterns the analysis was undertaken using MATLAB and R scripts written by the author (Supplementary Material), which conduct spatial randomization and then calculate mean slope (for surfaces) and mean distance (for point patterns).

## 3. Results

### 3.1. Surface Patterns

**Figure 5:**
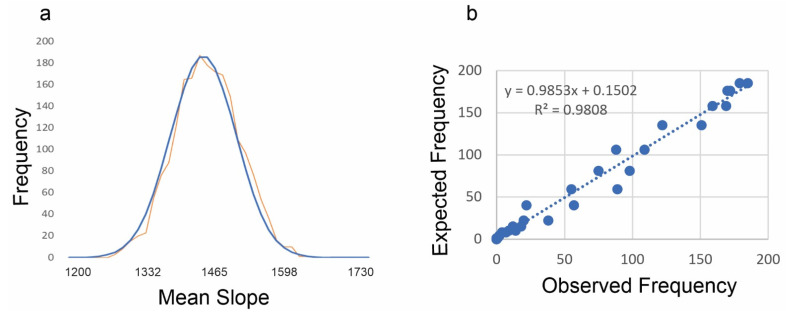
Match between the permuted mean slope and a normal distribution with mean and standard deviation equal to those of the permuted distribution: (**a**) overlay of the observed (orange) frequency on the expected frequency (blue); (**b**) linear fit between the observed and expected frequency across the distribution of mean slope. The x-axis in a is mean slope × 1000.

There was a perfect match between the distribution of mean slope of permuted surfaces and a normal distribution, as expected if the method is generalizable to surfaces (Figure 5). At 1,000,000 permutations the simulated distribution is very closely aligned with the Gaussian function with the same mean and standard deviation (Figure 5). This shows that the method produces a normal distribution of microstates, which is one of the key criteria identified in [10] to confirm the thermodynamic consistency of the method.

**Figure 6:**
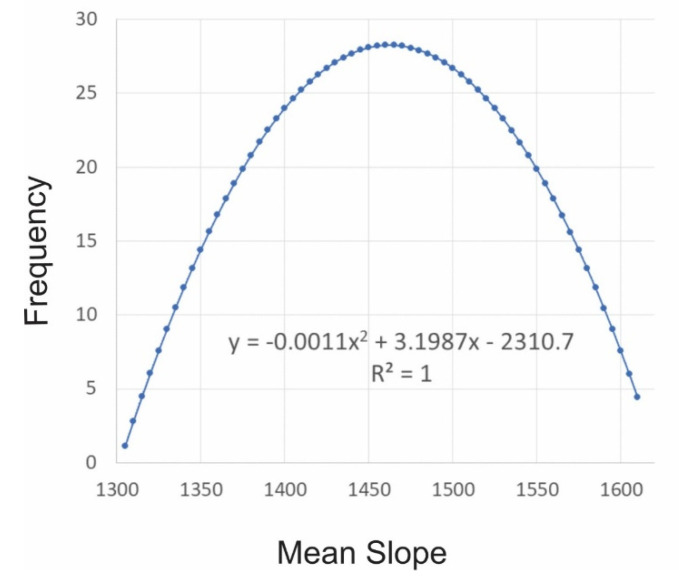
The entropy function for surface patterns shown in Figure 2. In the equation for the parabola y is the entropy (lnW) and x is the observed sum of slope across the surface. The x-axis is mean slope × 1000.

I computed the entropy function of this distribution of microstates across macrostate-space as in [4] by computing the log of the normal distribution, producing a parabolic entropy function (Figure 6). I tested this in a large number of permutations of the surfaces (1 × 10^10^) and the relationship was a perfect fit to a parabolic relationship (power 2), explaining 100% of the variance in the logarithm of the number of microstates in every level of the macrostate of mean slope (Figure 6).

**Figure 7:**
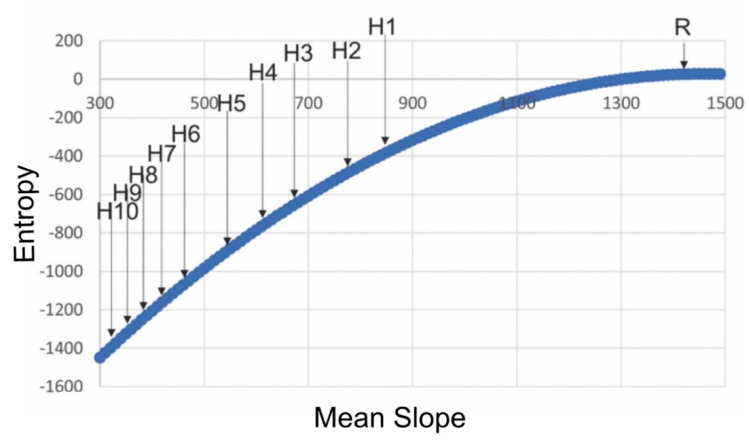
Plotting the spatial entropies of the 11 example surfaces from Figure 2 on the surface entropy function from Figure 6. The x-axis is mean slope × 1000.

I computed the entropy (lnW) for the 11 surface patterns (spatially random and the 10 levels of H aggregation; Figure 7). This showed, consistent with expectation and the performance of the method on landscape mosaics [3,4], that entropy is highest for random surfaces and is progressively lower for more aggregated patterns (Figure 7). This confirms that the Cushman method of direct application of the Boltzmann relation is fully generalizable to surfaces.

### 3.2. Point Patterns

**Figure 8:**
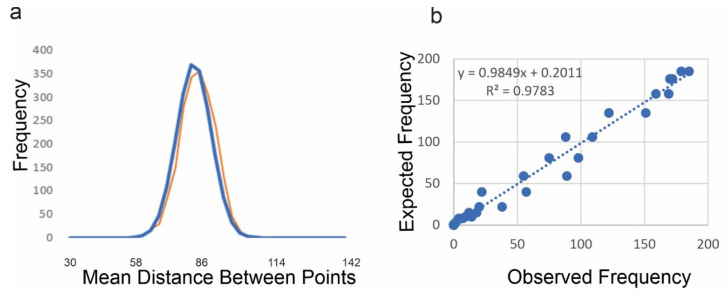
Match between the number of neighboring points connected by an edge distance of 1 in the permuted distribution of point patterns: (**a**) overlay of the observed (orange) frequency on the expected frequency (blue); (**b**) linear fit between the observed and expected frequency across the distribution of mean slope.

For point patterns I also found a very close match between the mean distance between points and a normal distribution across the permuted point patterns (Figure 8). This shows that the method produces a normal distribution of microstates for point patterns, and for lattices and surfaces, which suggests the method is thermodynamically consistent and generalizable across all three kinds of landscape pattern [10].

**Figure 9:**
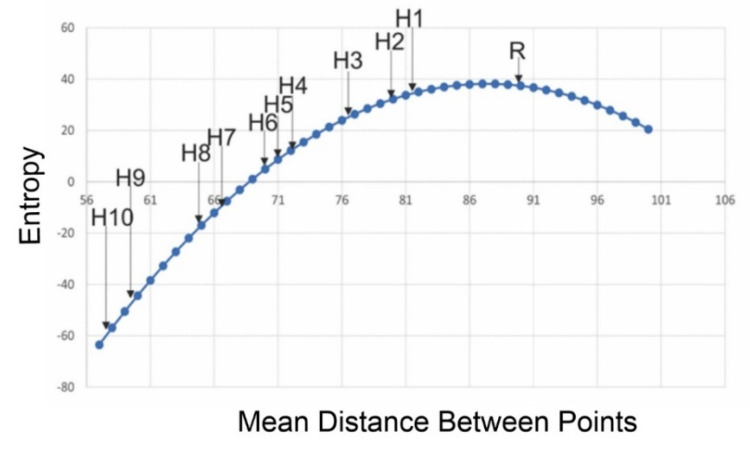
Plotting the spatial entropies of the 11 example point patterns from Figure 3 on the point pattern entropy function.

Similar to the surface pattern analysis, I fit the entropy function across 1 × 10^10^ permutations and confirmed it was also a perfect fit to a parabolic function. I then plotted the entropies of the 11 simulated point patterns across this entropy function (Figure 9). The simulated point patterns perfectly fall along the entropy curve in the expected pattern, with the spatially random distribution at the peak of the entropy function (maximum entropy) and the more aggregated point patterns progressively lower in entropy. This showed, consistent with expectation and the performance of the method on landscape mosaics [3,4], that entropy is highest for random point patterns and is progressively lower for more aggregated patterns (Figure 9). This confirms that the Cushman method of direct application of the Boltzmann relation is fully generalizable to point patterns, in addition to landscape mosaics and surfaces.

## 4. Discussion

Demonstrating that a single method to compute spatial configurational entropy is thermodynamically consistent and applicable to all spatial patterns is fundamentally important to provide a foundation for thermodynamic landscape ecology [10]. Until the present there have been relatively few explorations of the thermodynamic consistency and generalizability of spatial entropy methods. Cushman [3] proposed the direct application of the Boltzmann relation to computing configurational entropy in an attempt to be as faithful as possible to original theory with minimal modifications or additional assumptions. Cushman [4] also showed that this application is feasible based on the normal distribution of microstates and the parabolic entropy function, facilitating application to landscape mosaics of any number of classes or extent in pixels. Until the present, however, it had not been demonstrated that the method was thermodynamically fully consistent or extensible to all kinds of landscape patterns. Cushman [10] evaluated the thermodynamic consistency of the method and found that it met the three criteria of normal distribution of microstates, parabolic function of entropy, and monotonic increase in entropy in random mixing experiments from both aggregated and dispersed starting conditions. This paper takes the next step to generalize the Cushman method of computing configurational entropy to all kinds of landscape patterns, which include landscape mosaics, points patterns, and surfaces [12].

There are six important insights produced from this analysis. First, and most importantly, this analysis shows that the Cushman method of direct application of the Boltzmann relation to compute configurational entropy is directly and fully applicable to landscape mosaics, point patterns, and surfaces. The entropy of any landscape pattern can be calculated with the same theory, algorithm, and method. The only difference is the state variable used, which differs slightly among mosaics, surfaces, and point patterns. However, the state variable is fully analogous between them and conceptually equivalent within the physiognomy of the different landscape models. Specifically, the state variable for computing the entropy of a landscape mosaic is edge length; for surface patterns it is mean local slope; for point patterns it is the mean distance between pairs of points. Other than the state variable, the method is entirely the same between these approaches.

Second, this analysis shows that for any landscape pattern from any landscape conceptual model (sensu [12]) the entropy is readily calculated using the Boltzmann relation based on the sum of neighbor differences compared to the distribution of the sum of neighbor differences. As noted above, the only difference is the state variable used to calculate neighbor differences (edge, slope, or distance). For mosaics the sum of neighbor differences is the amount of edge. For mosaics the sum of neighbor differences is mean slope. For point patterns it is mean distance between points.

Third, in each case the number of microstates across the distribution of macrostates is normally distributed. Fourth, this analysis confirms the entropy function is parabolic. Fifth, it also confirms that maximum that entropy is reached at spatial randomness, for any kind of spatial pattern, including surfaces, point patterns, or patch mosaics. Sixth, the parabolic entropy function and the values of entropy calculated for the simulated landscape patterns confirm that entropy is minimized in extreme aggregation or dispersion.

Based on its consistency with fundamental theory (the original Boltzmann relation), its full thermodynamic consistency [10], and its generalizability to all kinds of landscape patterns, the Cushman approach for computing the configurational entropy of landscape patterns would seem to be potentially useful as a foundation for entropy research in landscape ecology. There have been several other approaches proposed to compute configurational entropy. The Gao [5,6] method has developed over several modifications into an approach that uses multi-resolution aggregation to compute Boltzmann entropy. This method has been implemented in an analytical tool, making it the first method that is readily available to practitioners who are not themselves technical experts in spatial data and permutational methods [13]. It has also been shown to be partly thermodynamically consistent and is applicable to both surfaces and landscape mosaics [9]. Similarly, there has been recent interest in applications of the Wassenstein formulation of Boltzmann entropy to computing the entropy of spatial patterns [7,8]. This method has not yet been fully evaluated for thermodynamic consistency and generalizability across different kinds of spatial patterns. More work is needed to compare the performance of these and other new approaches to computing landscape entropy. Future work should evaluate their similarities and differences across controlled gradients of pattern in mosaics, surfaces and point patterns to confirm their thermodynamic consistency and full generalizability.

It is often said that science should produce theories that are as simple as possible, but not simpler. Parsimony is an important principle of science and reflects fundamental attributes of nature. The simplest theory that explains the data is usually the best, not only in terms of heuristic value but also in matching ontological reality [14]. The astronomical model of Ptolemy seemed to match empirical observations as well as that of Copernicus, but with many more parameters requiring convoluted theory. In that case the elegance of theoretical simplicity matched reality. Similarly, the direct application of the simplest version of the original and iconic Boltzmann relation to spatial patterns seems to provide a parsimonious, consistent, generalizable, and interpretable measure of spatial entropy.

Of course, the development of a generalized metric for calculating configurational entropy of a landscape is an evolutionary rather than revolutionary advance; it is hardly a Copernican moment. However, I strongly believe that putting landscape ecology into a theoretical framework based on thermodynamics and entropy will prove to be revolutionary and transformative, and that quantitative methods to robustly calculate configurational entropy of any landscape model are essential to that effort. The connections between calculating landscape configurational entropy and classic thermodynamics are at several levels. First, conceptually using entropy relation to describe patterns in terms of entropy is a useful foundation for exploring more formal thermodynamic pattern–process relationships at the landscape level. Second, this method does not demonstrate that landscape pattern entropy is directly linked to formal thermodynamic processes, such as energy flow, emergence, and maintenance of dissipative structures. However, generalized and consistent methods of computing landscape configurational entropy are the foundation for taking this next step to explore linkages between energetic entropy and structural entropy at landscape scales.

There have been many efforts over the last five decades to link ecological theory with thermodynamics and entropy [2]. In the 1960s geomorphology and hydrology was first cast into the context of entropy, and methods and theories developed to describe landscape evolution as a process of entropy maximization to achieve the most probable distribution of energy and matter [15]. The calculation of configurational entropy of landscapes is conceptually closely linked to concepts of landscape evolution in geomorphology, and linking these branches of research is likely to be very fruitful. Specifically, the applications of entropy in hydrology and geomorphology have focused on developing predictions of the most likely condition of the system given its composition and constraints, and considered that most likely state to maximize entropy, and evaluated departure from that maximum entropy state. This is conceptually the same as that attempted by the different methods to calculate configurational entropy, which is to evaluate current landscape conditions relative to the most likely condition, based on the distribution of microstates.

An important topic for further exploration is that not all microstates of landscape configuration are likely to be equally probable, given constraints on landscape evolution due to gravitational, tectonic, erosive, hydrological, and vegetative processes, and anthropogenic factors. This suggests future work should explore modifications of the direct application of the Boltzmann relation to consider the unequal probability of different microstates, using, for example, Gibbs entropy instead of the Boltzmann relation. The challenge then becomes estimating the different probabilities of all possible microstates, which requires understanding and simulating complex space–time processes, which is a vastly more difficult task than using the assumption of equal probability of microstates and the Boltzmann relation as a null model.

One of the areas in which applications of entropy methods and concepts have been most widely developed in recent years is in work related to the Maximum Entropy Theory of Ecology [16], which is considered to be an entropy-based theory of distribution, abundance, and energetics. METE has largely used information entropy methods to evaluate the structure of ecological systems under the theory that ecosystems evolve to maximize entropy. It would be particularly useful to pursue work to integrate spatially-explicit measures of landscape entropy into evaluations of ecological complexity, macroecology, and the Maximum Entropy Theory of Ecology, given that the structure, function, and evolution of ecosystems are deeply influenced by pattern–process relationships at a range of scales [17]. Spatial configuration is a critical attribute of ecosystems, and any theory of ecology based on entropy therefore should formally engage and employ measures of configurational entropy within its analyses and theories.

**Figure 10:**
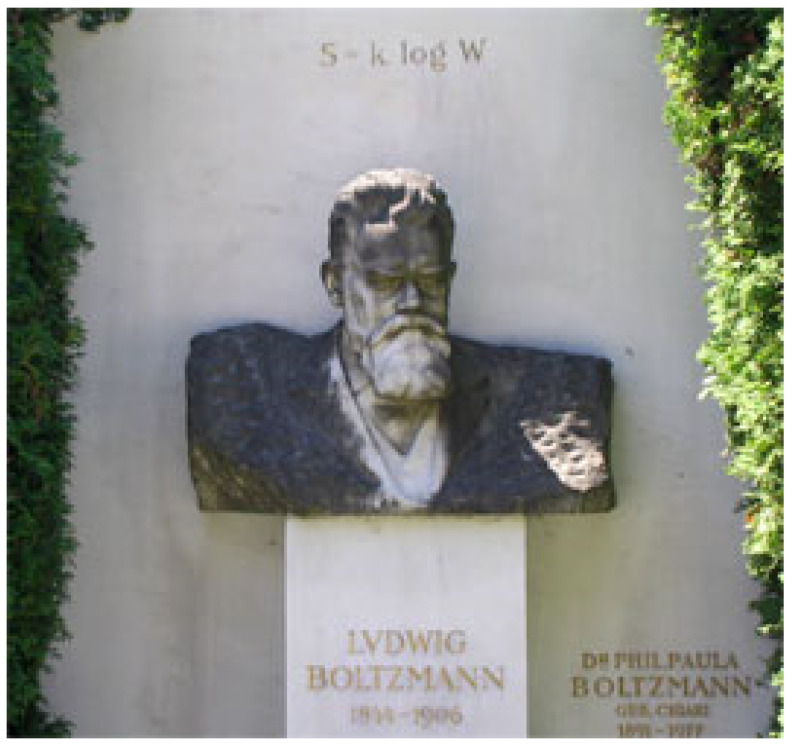
Boltzmann’s tomb.

The emergence of structure and order at a landscape scale is produced through the dynamics of dissipative structures [1,18,19]. Indeed, ecosystems are best considered to be spatially dependent networks of self-replicating dissipative structures. Integrating spatial measures of landscape entropy with thermodynamic analysis of ecosystem structure and energetics appears to be important to advancing and generalizing thermodynamic ecological research. I end with a quote from Boltzmann which encapsulates the profound simplicity of the second law as the arbiter of nature: “We have discovered the statistical way in which systems evolve. We see that the irreversibility of natural change results not from certainty but from probability. All structures and events correspond to the evolution of the Universe through successive states of increasing probability” (Figure 10).

## Data Availability

The data presented in this study are available in the Supplementary Material.

## Supplementary Materials

The following are available online at https://www.mdpi.com/article/10.3390/e23121616/s1.
